# Supplementary material for: Thymic Carcinoma: Unraveling Neuroendocrine Differentiation and Epithelial Cell Identity Loss
Source: Cancers (Basel). 2023 Dec 25;16(1):115. doi: 10.3390/cancers16010115 (PMC10778300; doi:10.3390/cancers16010115)
Supplement: Supplementary file 1 [file cancers-16-00115-s001.zip › cancers-2720519-supplementary.pdf]

Supplementary figure and figure legends for:

Thymic Carcinoma: Unraveling Neuroendocrine Differentiation and Epithelial Cell Identity Loss

Yosuke Yamada [1,*], Kosuke Iwane [2], Yuki Nakanishi [2] and Hironori Haga [1]

[1] Department of Diagnostic Pathology, Kyoto University Hospital, Kyoto 606-8507, Japan; haga@kuhp.kyoto-u.ac.jp

[2] Department of Gastroenterology and Hepatology, Kyoto University Graduate School of Medicine, Kyoto 606-8507, Japan; iwanekosuke@kuhp.kyoto-u.ac.jp (K.I.); yuki@kuhp.kyoto-u.ac.jp (Y.N.)

* Correspondence: yyamada@kuhp.kyoto-u.ac.jp; Tel.: +81-75-751-4946

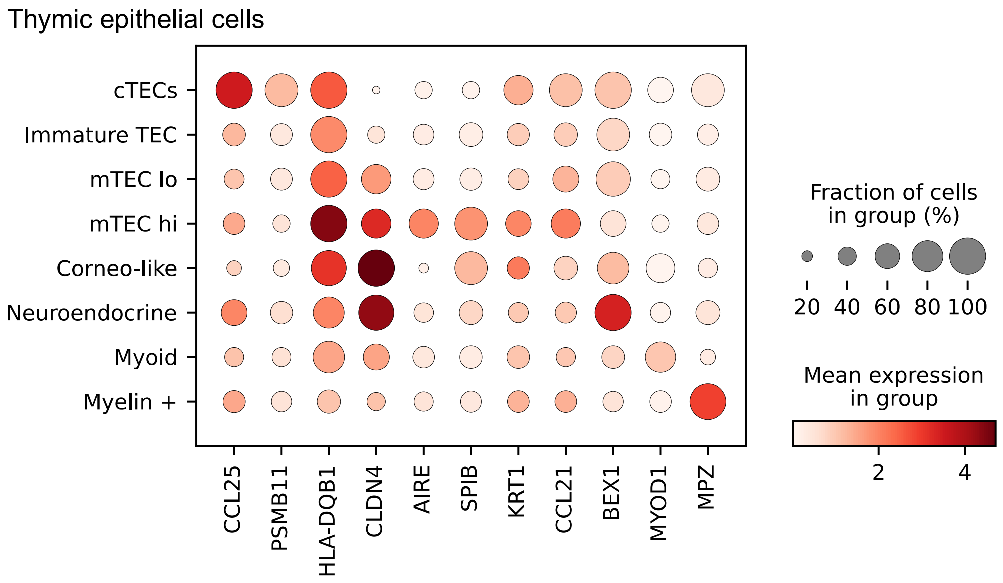

Figure S1. Expression of marker genes in each group. Expression patterns of marker genes in the eight thymic epithelial cell groups: cTECs, immature TEC, mTEC lo [low], mTEC hi [high], corneo-like, neuroendocrine, myoid, and myelin + (Bautista et al., 2021. [22] GSE147520).

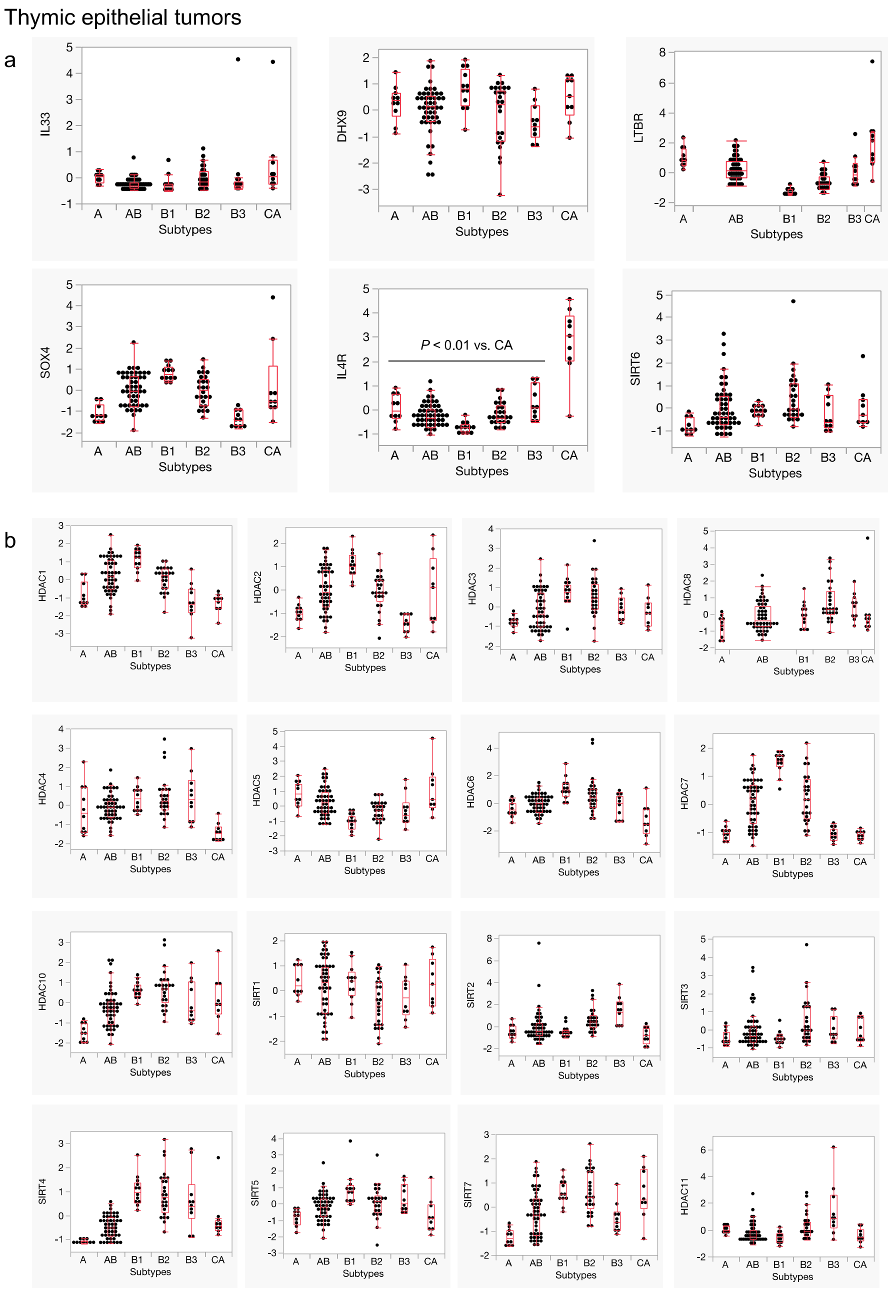

Figure S2. Expression of potential regulators of thymic tuft cell development and HDAC families in thymic epithelial tumors. (a) Among candidate genes involved in thymic tuft cell development, IL4R (in addition to HIPK2, HDAC9, FEZF2 [Figure 4]) exhibited significant expression in thymic carcinoma compared to thymomas ($P < 0.01$ for all thymoma subtypes vs. thymic carcinoma, Wilcoxon test). Expression levels of IL33, DHX9, LTBR, SOX4, and SIRT6 did not show remarkable differences among TET subtypes. (b) Expression of all HDACs other than HDAC9 in thymic epithelial tumors (TETs). Unlike HDAC9 (Figure 5), the other HDACs did not demonstrate subtype-dependent expression patterns (a, b: Thymoma, TCGA PanCancer Atlas [Y-axis: RNA-seq, Z score]).

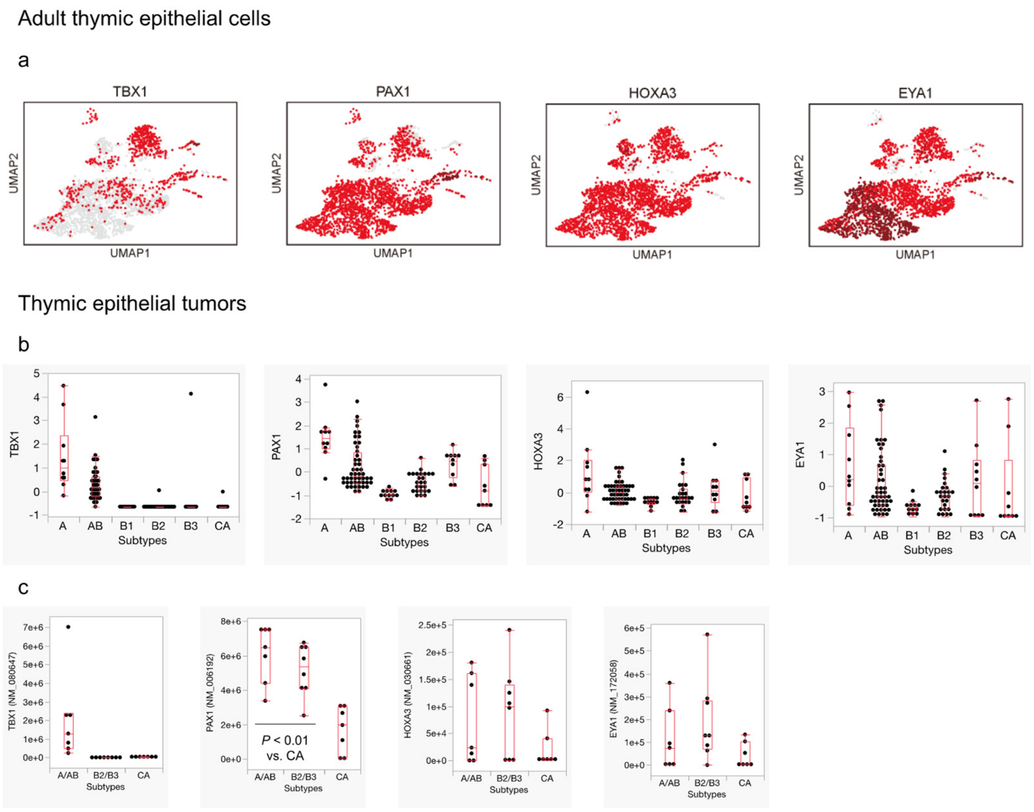

Figure S3. Expression of genes related to thymic organogenesis in adult thymic epithelial cells and thymic epithelial tumors. (a) TBX1, PAX1, HOXA3, and EYA1 are expressed in most adult thymic epithelial cells, indicating their status as pan-thymic epithelium markers. (b,c) Thymic carcinoma tends to express the above genes at lower levels than thymomas. The difference in PAX1 expression level between thymomas and thymic carcinoma is significant in the dataset by Petrini et al. [34] ($P < 0.01$, Wilcoxon test). (a: Bautista et al. [22], 2021. GSE147520; b: Thymoma, TCGA PanCancer Atlas [Y-axis: RNA-seq, Z score]; c: Petrini et al., 2014 [34] [Y-axis: RNA-seq, FPKM]).
